# Evaluation of the Effectiveness of Different Types of Professional Tooth Whitening: A Systematic Review

**DOI:** 10.3390/bioengineering11121178

**Published:** 2024-11-21

**Authors:** Andrea Butera, Carolina Maiorani, Gitana Rederiene, Stefano Checchi, Gianna Maria Nardi

**Affiliations:** 1 Unit of Dental Hygiene, Section of Dentistry, Department of Clinical, Surgical, Diagnostic and Pediatric Sciences, University of Pavia, 27100 Pavia, Italy; 2 European Dental Hygienists Federation, Groenewoudsedijk 40, 3528 BK Utrecht, The Netherlands; grederiene@gmail.com; 3 Vilnius University Hospital Zalgirio Clinic, 08217 Vilnius, Lithuania; 4 Dental Hygiene Degree Course, Department of Surgical Sciences, Dental School, University of Turin, 10126 Turin, Italy; stefano.checchi@unito.it; 5 Department of Odontostomatological and Maxillofacial Sciences, Sapienza University of Rome, 00161 Rome, Italy; giannamaria.nardi@uniroma1.it

**Keywords:** dental bleaching, hydrogen peroxide, carbamide peroxide, dental discoloration, biomaterials, dental health

## Abstract

Background/Objectives: Tooth whitening is a cosmetic dental treatment that improves the color of natural teeth, making them whiter and brighter; this review aimed to evaluate the greater effectiveness of in-office, at-home, and combined bleaching with hydrogen or carbamide peroxide, also in relation to possible relapses and side effects (tooth sensitivity). Methods: A literature search has been carried out through electronic databases, PubMed/MEDLINE, and Cochrane Library, focused on the use of the effectiveness of in-office, at-home, and combined dental bleaching. This review has been registered on PROSPERO (ID613248). Results: 30 articles have been included. Most of the studies did not find any more effective treatment than the proposed treatments; all types of bleaching have been shown to be effective in changing color; in the studies that have compared in-office and at-home bleaching, there was a lower recurrence of treatment with the use of the at-home trays with carbamide peroxide gel. There were no clear positions regarding tooth sensitivity, which would appear to be higher in professional in-office and combined dental bleaching. Conclusions: Bleaching is an effective treatment for the change of color of the tooth regardless of the type used (concentrations, type of gel, and duration of sessions), although, at home, it would seem to be more effective over time. It is, however, a clinical feature that may give a risk of tooth sensitivity after treatment.

## 1. Introduction

The WHO defines health as a state of complete physical, mental, and social well-being. A beautiful, healthy smile is undoubtedly an expression of psychosomatic well-being [1]. In common culture, a smile represents happiness and is used to communicate and connect with others, influencing not only beauty standards but also complex areas such as self-esteem. Often, inner qualities and behaviors are influenced or accentuated by aesthetic features [2,3]. Teeth are not only essential for chewing but also play a crucial role in speech and social interactions: An inability to smile due to poor oral health can significantly limit one’s social relationships and affect overall social life [4,5].

Aesthetic dentistry and the perception of one’s smile have gained increasing importance within the population. The aesthetic appeal of a smile has become a greater psychological and social concern, with dental appearance and disorders often impacting self-esteem and self-perception [6,7,8]. Many individuals wish to change aspects of their smile, particularly the color of their teeth, which can cause discomfort, embarrassment, and anxiety in social contexts [5,9].

The color of a tooth is primarily determined by the underlying dentin [10,11,12]. Tooth shape also plays a role, as smooth and even surfaces appear brighter, while irregular surfaces reflect less light and appear more translucent [13]. Translucency or opacity is determined by enamel composition, consisting of organic matter, inorganic particles, and water [14,15,16]. Teeth are more translucent if crystal structures predominate, while a higher organic content makes them opaquer and whiter [14,16,17,18]. Enamel-rich teeth often appear brighter and less translucent [13]. Over time, tooth color can change as the enamel wears down, making dentin more visible and leading to a more orange hue with age [13,14,15,19].

Various factors cause changes in tooth color, including extrinsic and intrinsic discolorations. Extrinsic discoloration results from substances that come into contact with tooth surfaces—such as coffee, tea, alcohol, smoking, poor oral hygiene, or certain ingredients in toothpaste or mouthwash (e.g., chlorhexidine) [19,20,21,22,23,24]. Sodium bicarbonate-based powders are effective for removing stains but do not lighten the color of the tooth [25]. In contrast, intrinsic discoloration relates to internal factors like prolonged fluoride intake, genetic mutations, vitamin deficiencies, trauma, cavities, and amalgam restorations [26,27,28,29,30,31,32].

Tooth whitening can address extrinsic discoloration and age-related changes but not intrinsic discoloration. This cosmetic dental treatment lightens natural teeth, making them appear whiter and brighter [19]. The two primary bleaching treatments are in-office and at-home, both using active agents: hydrogen peroxide and carbamide peroxide. These agents release oxygen molecules upon contact with tooth tissues, penetrating the enamel to break down pigment molecules responsible for stains [33,34]. These compounds have been shown to be safe at the systemic level, with minimal local side effects [35,36,37].

Professional tooth whitening involves applying a gel with 25–40% hydrogen peroxide or up to 37% carbamide peroxide, activated by LED or laser lights or self-activated. This treatment typically follows a professional hygiene session that removes bacterial biofilm and calculus to allow proper tissue healing [19,34,38,39,40,41]. The gums are protected with a mouth gag and rubber dam during in-office procedures, though techniques vary by manufacturer. Patients are advised to follow specific dietary and lifestyle guidelines for several days after in-office whitening [38,39,40,42].

At-home whitening involves custom-made trays created from dental impressions, worn either during the day or at night with a gel containing 6% hydrogen peroxide or 10–16% carbamide peroxide [19]. Treatment duration varies by manufacturer and time of use (day or night). It is recommended to perform whitening after a professional hygiene session under the guidance of a dental professional [41,43].

Both professional and at-home whitening require consultation with a dentist or dental hygienist to assess suitability. An evaluation of smile aesthetics and tooth color should precede treatment, as intrinsic discolorations, enamel defects, and genetic conditions (e.g., amelogenesis and dentinogenesis imperfecta), fluorosis, trauma, or staining from old restorations cannot be effectively treated with bleaching alone [44,45].

A color assessment using an intraoral scanner or spectrophotometer should be performed to determine hue (base color on an A–B–C–D scale), chroma (saturation level, influenced by age, and enamel thickness), and lightness (brightness). This assessment guides the choice of the appropriate whitening method [46,47]. Whitening can sometimes cause temporary sensitivity, which suggests inflammatory changes in the dental pulp, though the exact cause remains unclear. It may result from fluid movement within the dentinal tubules, stimulating pulpal receptors, or from surface alterations caused by low pH and chelating agents [48,49,50,51,52]. Another common issue is gum irritation, which can cause redness, swelling, or white spots when the gel contacts gingival tissue [48]. In these cases, treatment should be paused and antiseptic or soothing gels or mouthwashes with natural antiseptics applied [53,54,55].

However, the relative effectiveness of professional versus at-home whitening remains unclear. This review aims to assess, through literature analysis, whether one treatment—either in-office or at-home—is more effective, particularly in terms of relapse rates and side effects such as tooth sensitivity.

## 2. Materials and Methods

### 2.1. Focused Question

Evaluate the effectiveness of in-office and/or at-home dental bleaching (primary outcome), including relapses and side effects (secondary outcome).

### 2.2. Eligibility Criteria

The following inclusion criteria guided the analysis of the studies:

Type of studies. Randomized clinical trial, blind/double-blind clinical trial, case-control, cross-sectional, cohort studies, observational studies, and in vitro studies.

Type of participants. Healthy patients over 18 undergoing professional in-office and/or at-home dental bleaching.

Type of interventions. Professional in-office and/or at-home dental bleaching with hydrogen peroxide (HP) or carbamide peroxide (CP).

Type of results. Determination of the effectiveness of the type of bleaching, including consideration of side effects and relapse.

Only studies that met all the inclusion criteria were included. However, the following exclusion criteria were considered: articles published before 2014 and reviews.

### 2.3. Search Strategy

The population, intervention, comparison, outcome (PICO) model was used to perform this systematic review through examination of studies identified in electronic databases, PubMed/MEDLINE, and Cochrane Library. Initially, all study abstracts were taken into consideration, and all studies that met the inclusion criteria and evaluated efficacy of dental bleaching (in-office or at-home) were reviewed and analyzed. This review has been registered on PROSPERO (ID613248).

We performed the search using the following keywords: dental bleaching, dental whitening, professional dental bleaching, professional dental whitening, in-office dental bleaching, at-home dental bleaching, combined dental bleaching, in-office AND at-home dental bleaching, in-office dental whitening, at-home dental whitening, combined dental bleaching, in-office AND at-home dental bleaching, in-office professional dental bleaching, at-home professional dental bleaching, combined professional dental bleaching, in-office AND at-home professional dental bleaching, in-office professional dental whitening, at-home professional dental whitening, combined professional dental whitening, and in-office AND at-home professional dental whitening.

### 2.4. Screening and Selection of Articles

A keyword search initially yielded 571 results, covering all authors. Duplicate records from multiple searches were identified and removed by a single author.

In the first phase, results were filtered to focus on professional in-office and/or at-home dental bleaching studies. Studies that did not meet the eligibility criteria, such as reviews, meta-analyses, and studies not published in English, were excluded.

**Figure 1:**
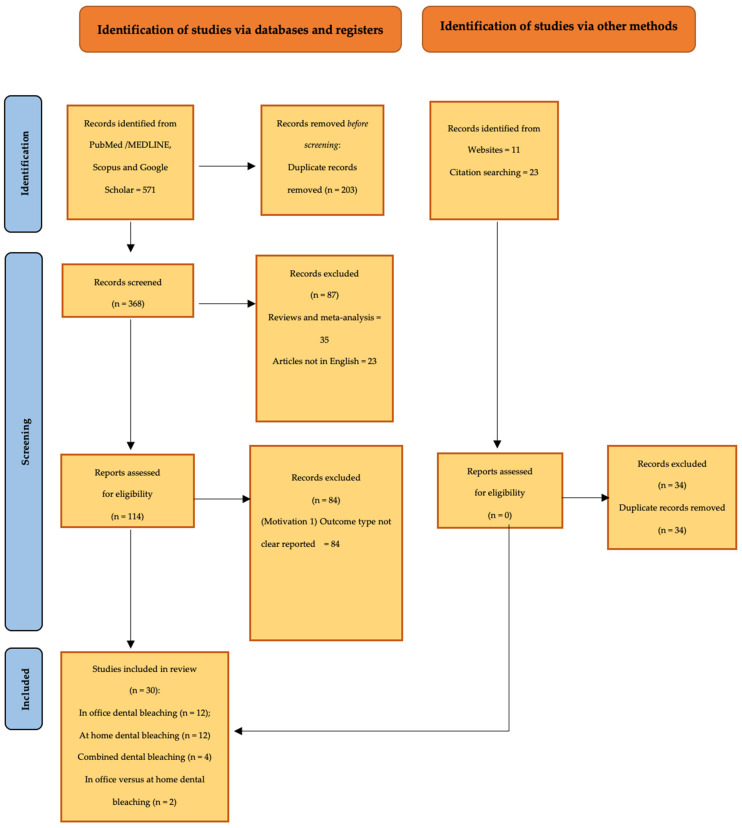
Flow chart of included studies: From 571 articles, duplicates were eliminated, and 368 articles have remained; from the first analysis of the abstracts, some articles were deleted that did not coincide with the eligibility criteria, and 114 reports remained for full readings of articles. After reading and according to the criteria, included in review were 30 articles.

The same authors then proceeded to examine the remaining articles in full-text format (Figure 1).

## 3. Results

### 3.1. In-Office Dental Bleaching

Methods and participants. The 12 studies selected for this review are published in English: Most of them are clinical trials or randomized clinical trials where patients have undergone one or more professional dental bleaching sessions in-office of different durations. In the studies involved, a total of 656 patients were analyzed (one study was conducted in vitro) [56] with a follow-up of 14 days (8.3%) [57], 1 month (25%) [58,59,60], 3 months (8.3%) [61], 6 months (25%) [62,63,64], 1 year (16.7%) [65,66], and 3 years (8.3%) [67].

Intervention. The bleaching substances used in different percentages were HP and CP. A total of 58.3% of the studies used HP 35% [56,60,62,63,66,67], 16.7% HP 37.5 [59,65]-25 [56,67]-6% [59,65] and CP 35% [57,63], 8.3% HP 40 [56]-38 [58]-18% [56], and CP 37% [66]; 50% of the studies used LED light [57,61,63,64,66,67]. Tooth sensitivity was assessed in 58.3% of the studies [57,58,60,61,63,64,67], and one study also assessed gum irritation [60].

Outcomes. The tested products demonstrated effectiveness in improving tooth color regardless of the concentration used or the number and timing of sessions. Some studies indicated greater effectiveness with higher concentrations compared to lower ones [56,59,65]. In terms of tooth sensitivity, the studies reviewed did not reveal significant differences based on the type of treatment applied; however, in studies that incorporated LED light, post-treatment sensitivity appeared reduced [57,61,63,64,67]. While LED light does not seem to significantly impact the efficacy of whitening (measured by color change), one study reported enhanced whitening effectiveness when LED light was used [57] (Table 1).

### 3.2. At-Home Dental Bleaching

Methods and participants. The 12 studies selected for this review are published in English: Most of them are clinical trials or randomized clinical trials where patients have undergone at-home dental bleaching of different durations. In the studies involved, a total of 755 patients were analyzed with a follow-up of 5 weeks (8.3%) [68], 1 month (25%) [41,69,70], 3 months (8.3%) [71], 6 months (16.7%) [72,73], 30 months (8.3%) [74], 42 months (8.3%) [75], and 1 year (25%) [76,77,78].

Intervention. The bleaching substances used in different percentages were HP and CP. A total of 58.3% of the studies used CP 10% [41,68,72,73,74,76,77], 33.3% HP 10% [69,70,76,78], 16.7% HP 6% [72,78], 8.3% HP 4 [70]-6.5 [71]-7.5% [78], and CP 16 [75]-37% [41]. Tooth sensitivity was assessed in 50% of the studies [61,68,69,70,71,76], and two studies also assessed gum irritation [41,76].

Outcomes. The use of the tested products has demonstrated effectiveness in improving color regardless of concentration or treatment duration. One study found greater effectiveness with 10% carbamide peroxide (CP) compared to 6% hydrogen peroxide (HP) [72]. No significant differences were observed in tooth sensitivity after whitening, except in one study where higher gel concentrations led to more side effects [70] and another study where longer treatment durations were associated with the onset of similar effects [71] (Table 2).

### 3.3. Combined Dental Bleaching

Methods and participants. The four studies selected for this review are published in English: Most of them are clinical trials where patients have undergone one professional dental bleaching session in an office or at-home dental bleaching compared with the use of both bleaching systems. In the studies involved, a total of 223 patients were analyzed with a follow-up of 14 days (25%) [79], 43 days (25%) [80], 4 weeks (25%) [81], and 6 months (25%) [82].

Intervention. The bleaching substances used in different percentages were HP and CP. In a study, the use of HP 35% in office was compared with the combined use of HP 35% in office and HP 6% at home [79]; in a study, the use of HP 40% in office was compared with CP 10% at home and compared with the combined use of HP 40% in office and CP 10% at home [80]; in a study, the use of HP 40% in office was compared with HP 10% at home and compared with the combined use of HP 40% in office and HP 10% at home [81]; in a study, the use of HP 38% in office was compared with the combined use of HP 38% in office and CP 10% at home [82]. Tooth sensitivity was assessed in all studies, and one study also assessed gum irritation [81].

Outcomes. The studies analyzed show mixed results. It appears there is no significant difference between combined bleaching and using either in-office or at-home bleaching alone, with only one study reporting better results for combined bleaching. One study found increased tooth sensitivity with the use of combined or in-office bleaching [80], while another reported greater sensitivity with combined and at-home bleaching treatments [81] (Table 3). 

### 3.4. In-Office Versus At-Home Dental Bleaching

Methods and participants. The two studies selected for this review are published in English: Most of them are clinical trials or randomized clinical trials where patients have undergone one professional dental bleaching session in-office or at-home dental bleaching. In the studies involved, a total of 50 patients were analyzed with a follow-up of 6 months.

Intervention. The bleaching substances used in different percentages were HP and CP. In a study, the use of HP 35% in-office was compared with CP 16% at home [83]; in the other study, the use of HP 38% in-office was compared with CP 15% at home [84].

Outcomes. The studies indicate that in-office dental bleaching is associated with a higher recurrence rate compared to at-home treatments and also causes greater tooth sensitivity (Table 4).

### 3.5. Risk of Bias

Randomization, allocation concealment, blinding, outcome data, and outcome recording were assessed. A color code was assigned based on the level of risk: aAgreen symbol was used when the information was complete for the considered variable (low risk of bias); a yellow symbol was used when information was missing or unclear (moderate risk of bias); and a red symbol was assigned when the procedure was not properly executed (high risk of bias) [85].

Table 5, Table 6, Table 7 and Table 8 show the risk of bias in the main articles examined; this review presents a moderate risk of bias.

## 4. Discussion

The objective of this review was to assess the effectiveness of in-office, at-home, and combined dental bleaching treatments. Additionally, where applicable, the long-term effects on color stability (measured using the Vita Classical, Vita Bleached guide, and Easyshade Spectrophotometer) and tooth sensitivity (evaluated using the Visual Analogue Scale) were also considered.

The studies included in this review evaluated patients of various ages who underwent dental bleaching treatments, whether in-office, at home, or through a combination of the two methods. The bleaching agents used in these treatments are those commonly applied in clinical practice, specifically hydrogen peroxide (HP) and carbamide peroxide (CP), with varying concentrations and protocols in terms of treatment duration and method of application. For in-office bleaching, HP was generally preferred, applied at concentrations from 4% to 40%, while CP was used in only 25% of cases. Sessions lasted between 20 and 40 min or, in some instances, up to five sessions of 15 to 45 min each at weekly intervals [56,58,59,60,61,62,63,64,65,66,67]. For at-home bleaching, CP was used at concentrations from 10% to 37% [41,68,71,72,73,74,75,76,77] or HP at 4% to 10%, with application times from 30 min to 10 h per day over a period of 7 to 28 days [69,70,71,72,76,78]. For combined bleaching, at-home treatment was conducted for 1 to 2 weeks, followed by a single in-office session [79,80,81,82].

The results indicate that all treatment types effectively achieve the desired clinical outcomes, with no one treatment proving more effective than the others. Studies comparing different treatment types showed mixed findings, consistent with prior research showing no significant differences in efficacy or tooth sensitivity for most cases [39,44]. Additionally, the findings in this review do not account for variability across clinical protocols, including differences in daily usage duration, number of sessions, and product concentrations across whitening techniques [86].

For in-office treatments, no significant differences in color change were observed regardless of product type (HP or CP at different concentrations), whether performed in single or multiple applications (up to four) with varying durations and intervals (typically weekly). Some studies suggest that higher concentrations of HP may be more effective for color change [56,59,65], and differences in tooth sensitivity may depend on the use of LED light as an adjunct [57,61,63,64,67]; indeed, LED light appears to enhance the effectiveness of whitening without increasing dentinal sensitivity [57]. However, this is not consistently supported in the literature, where no conclusive evidence confirms this effect [87,88]. Bersezio et al. (2019) and Estay et al. (2020) highlight that a higher concentration of HP (comparing HP 37.5% to HP 6%) is effective in achieving and maintaining color change over time without relapse [59,65]. This contrasts with Maran et al. (2020), who report that the concentration of HP, whether low or high, does not significantly impact results [39]. This review also indicates that a single session can be as effective as multiple sessions, with other studies confirming that additional gel applications do not necessarily improve color outcomes, although further research is needed [89].

In examining at-home bleaching, no significant differences were observed in treatment efficacy based on higher or lower concentrations or longer or shorter exposure times. However, some researchers argue that higher concentrations may improve whitening results, albeit with a greater risk of tooth sensitivity [70], as can extending treatment duration [73]. At-home bleaching appears to provide effective long-term results, lasting up to one year [77]. One study suggests a difference in efficacy related to the bleaching agent type [72], indicating that carbamide peroxide may be more effective, consistent with the existing literature [90].

Combined bleaching, however, does not appear to offer additional benefits over single-type treatments and may increase tooth sensitivity, according to studies in this review [80,81]. This finding is supported by other research comparing professional tooth whitening methods, which suggests that combined bleaching does not enhance color intensity beyond that achieved with single treatments [91]. A recent systematic review and meta-analysis similarly found no statistically significant differences in color outcomes between in-office and combined bleaching, though higher tooth sensitivity was associated with the combined approach [92].

Studies comparing approaches generally reported similar effectiveness, except in comparisons of at-home and in-office bleaching, where at-home treatments were associated with longer-lasting results [83]. Notably, though based on only two studies, the results suggest that in-office whitening with high HP concentrations may be linked to higher recurrence rates within six months compared to at-home treatments using CP [83,84]. This finding aligns with the recent literature indicating that color achieved with at-home bleaching remains stable for 1 to 2.5 years, though relapses may occur sooner in cases of severe discoloration [93].

Overall, it is important to highlight that, in studies where patient satisfaction [61,77,78], self-perception of whitening [57,60,61,81], or quality-of-life impacts and psychological well-being were assessed through questionnaires [60,61,63,65], patients reported positive feedback.

A limitation of this review is the heterogeneity of the studies, which prevents identifying a superior treatment type, even regarding side effects such as tooth sensitivity. The studies varied widely in terms of the whitening gels used, session durations (for in-office treatments), overall treatment durations (for at-home treatments), gel concentrations, and follow-up. Future studies would benefit from standardizing these variables to enable more meaningful comparisons.

New trends in dental whitening include the use of hybrid lights (LED/laser), which can reduce in-office treatment time and minimize associated dentinal sensitivity while maintaining the same efficacy in color change [67,94]. Additionally, gels (for both in-office and at-home whitening) containing titanium dioxide nanoparticles co-doped with nitrogen and activated by third-generation visible light show promise in achieving effective whitening with reduced dentinal sensitivity, though further clinical studies are needed to confirm these properties [36,95]. Investigating the efficacy of these alternative therapies, by comparing them with standard clinical protocols, could provide insights into which treatment offers the best outcomes regarding both efficacy and side-effect reduction.

## 5. Conclusions

Tooth whitening treatments use hydrogen peroxide and carbamide peroxide gels to restore the natural color of teeth by removing intrinsic and extrinsic stains. Available methods include in-office, at-home, and combined approaches, with effectiveness depending on the type of gel, its concentration, and application time. While all methods generally yield positive results, at-home bleaching with carbamide peroxide may provide longer-lasting whitening with fewer relapses compared to in-office treatments. However, in-office treatments using higher concentrations can initially be more effective, though they may carry a greater risk of side effects, such as increased sensitivity. Further research is necessary to validate these findings, as there is no definitive consensus on the optimal approach or on the sensitivity levels associated with each method.

## Figures and Tables

**Table 1:** Studies focused on in-office dental bleaching included in this review [56,57,58,59,60,61,62,63,64,65,66,67].

Authors	Type of Study	Problem	Intervention and Control	Measurements	Outcomes
Altınışık 2023 [56]	In vitro	Using varying hydrogen peroxide concentrations, assess how well in-office bleaching in aesthetic dentistry affects the enamel surface’s roughness and color stability.	50 human incisors were randomly divided into the following: Group 1: No bleaching was performed in the control group. Group 2: 40% HP with fluoride (F); 2 applications of 20 min for session (2 sessions 7 days apart). Group 3: 35% HP with calcium (Ca); 1 application of 40 min for session (2 sessions 7 days apart). Group 4: 25% HP with nano-hydroxyapatite (nHA); 3 applications of 15 min for session (2 sessions 7 days apart). Group 5: 18% HP with nHA; 5 applications of 10 min for session (2 sessions 7 days apart).	Color at baseline, after the first and second sessions of bleaching, and, finally, after the staining protocol (*Easyshade Spectrophotometer*).	The highest coloration after bleaching was seen with 40% HP with F and 25% HP with nHA, while the lowest was observed with 35% HP with Ca and 18% HP with nHA, with no significant difference compared to the control group.
Brugnera 2020 [57]	RCT	Examine the impact on tooth color change and sensitivity of the violet light-emitted diode (LED) light (405–410 nm) used in in-office dental bleaching with 35% carbamide peroxide.	50 patients were selected and randomized into the following: Group 1: CP 35% two bleaching sessions of 30 min each, with 7-day intervals; Group 2: CP 35% two bleaching sessions of 30 min each, with 7-day interval + LED.	Color at baseline and 14 days after the second session (*Vita Classical*, *Vita Bleached guide*, *and Easyshade Spectrophotometer*). Tooth sensitivity immediately after treatment and 48 h post-session (*Visual Analog Scale*).	The violet LED light (405–410 nm) could improve dental bleaching effectiveness without sensitivity increase.
Martins 2018 [58]	RCT	Examine the differences in tooth sensitivity and bleaching effectiveness between two 20 min treatments and a 40 min application of 38% hydrogen peroxide.	41 patients were divided into (split-mouth) the following: Group 1: HP 38% (2 × 20 min) Group 2: HP 38% (1 × 40 min)	Color at baseline and 30 days following the second treatment (*Vita Classical*, *Vita Bleached guide*, *and Easyshade Spectrophotometer*). Tooth sensitivity up to 48 h (*Visual Analog Scale*).	The use of a 40 min in-office bleaching agent gel application produced the same whitening degree and tooth sensitivity that the two 20 min bleaching agent applications did.
Bersezio 2019 [59]	RCT	Examine whether 37.5% and 6% hydrogen peroxide (HP) gel may produce results that are equally good.	33 patients were divided into (split-mouth) the following: Group 1: HP 37.5% (3 × 12 min) Group 2: HP 6% (3 × 12 min) Two sessions of bleaching were carried out each week.	Color at baseline, one week, and one month after treatment (*Vita Classical and Easyshade Spectrophotometer*).	Both groups showed color variation between the initial measurement and subsequent times. One-month post-treatment, ΔE was 9.06 in the 37.5% HP group and 5.69 in the 6% HP group, with a statistically significant difference beginning in the second session.
Favoreto 2024 [60]	RCT	Analyze the participants’ self-perception, color change, side effects, and impact on oral health after undergoing in-office dental bleaching.	165 patients were divided into the following: Group 1: HP 35% 2 applications of 20 min each (2 × 20 min) Group 2: HP 35% 1 × 40 min Group 3: HP 35% 1 × 30 min	Color at baseline, after first and second sessions, and 30 days after treatment (*Vita Classical*, *Vita Bleached guide*, *and Easyshade Spectrophotometer*). Tooth sensitivity and gingival irritation at 1, 24, and 48 h after treatment (*Visual Analog Scale*).	A similar color change was noted (*p* < 0.001), showing no significant difference among the groups. The group 2 × 20 min showed the greatest risk of TS (76%, 95% CI 63 to 85) when compared to the 1 × 30 min (*p* < 0.04). The severity of TS and GI along with the GI risk was comparable across the groups (*p* > 0.31).
Mayer-Santos 2022 [61]	RCT	Examine how bleaching using violet LED light (405–410 nm), either alone or in combination with hydrogen peroxide (HP) gel, affects color change and tooth sensitivity.	100 patients were divided into the following: Group 1: 35% HP (4 sessions, 1×/week) Group 2: violet LED (4 sessions, 1×/week) Group 3: violet LED (4 sessions, 2×/week) Group 4: hybrid technique (violet LED + HP 35%; 4 sessions, 1×/week)	Color at baseline, 14 days, and 3 months after treatment (*Colorimetric tests*). Tooth sensitivity (*Visual Analog Scale*).	Color variation was similar between techniques in G1 and G4, while G2 and G3 showed no difference but were less effective in bleaching. In terms of tooth sensitivity, G2 and G3 reported no sensitivity, and G4 showed lower sensitivity than G1.
Tsujimoto 2021 [62]	Clinical trial	Analyze how light exposure affects an in-office whitening agent’s therapeutic efficacy.	The in-office whitening agent (35%) was used in this study: Group 1: HP 35% with light irradiation (5 min + 3 min with lamp + 7 min for three times) Group 2: HP 35% without light irradiation (20 min)	Color at baseline, immediately after, and 6 months after treatment using a spectrophotometer (*Easyshade Spectrophotometer*).	The ΔE and shade had no significant differences with or without light irradiation.
Santos 2021 [63]	RCT	Examine the effects of an in-office violet light emitting diode (LED) system (405 nm) on tooth whitening, sensitivity, post-whitening medicine use, and overall quality of life.	80 patients were divided into the following: Group 1: violet LED (30 min) Group 2: CP 35% and violet LED (30 min); Group 3: only CP 35% (30 min) Group 4: HP 15% for 15 min for three sessions 4 whitening sessions, 7 days apart	Color at baseline, 15 and 180 days after treatment (*Vita Classical and Easyshade Spectrophotometer*). Tooth sensitivity at baseline, 15, and 180 days after treatment (*Visual Analog Scale*).	The findings indicated that the violet LED did not whiten teeth with the same intensity on its own as it did when combined with 35% CP. Nonetheless, since pain was not regularly noted in G2, it can be proposed that the treatment with LED + 35% CP resembles that of 35% HP for tooth whitening, albeit with superior pain results.
Sobral 2021 [64]	RCT	Evaluate the level of tooth sensitivity (TS), alterations in color, and treatment durability during a 6-month follow-up for two bleaching techniques.	60 patients were divided into the following: Group 1: one bleaching session with an HP 35% gel and a second bleaching session after 7 days (40 min for each session). Group 2: two bleaching sessions with two applications per session (30 min for one application), each session with a 7-day interval, using a light-emitting diode (LED) device emitting violet light (405–410 nm) without employing peroxide-containing bleaching gel.	Color at baseline, immediately after each bleaching session, and again at 15, 30, and 180 days. (*Vita Classical*, *Vita Bleached guide*). Tooth sensitivity before and after each bleaching session (*Visual Analog Scale*)	Violet light alone effectively bleached teeth and had the clinical advantage of causing less immediate postoperative sensitivity. However, it resulted in unwanted repigmentation after the bleaching treatment.
Estay 2020 [65]	Randomized and prospective double-blind clinical trial	In an in-office bleaching situation, compare the color change and stability of low-concentration (6%) hydrogen peroxide gel (HP) to that of typical 37.5% gel after a year.	25 patients: Group 1: HP 6% (3 × 12 min) Group 2: HP 37.5% (3 × 12 min) Two sessions of bleaching were carried out each week.	Color at baseline, following first session, 7 days, and 1 and 3 months after treatment (*Vita Classical*, *Vita Bleached guide*, *and Easyshade Spectrophotometer*).	After 1 year, 37.5% HP showed significantly better color rebound than 6% HP.
Kury 2022 [66]	RCT	Examine the long-term effects of bleaching in a workplace using violet LED light (LED) either by itself or in conjunction with hydrogen (HP) or carbamide (CP) peroxides.	82 patients were divided into the following: Group 1: LED, Group 2: CP 37% Group 3: LED/CP 37% Group 4: HP 35% Group 5: LED/HP 35% Three sessions at 7-day intervals (30 min)	Color at baseline, 6 and 12 months after treatment applying the CIELab coordinates’ values obtained using a spectrophotometer (*Easyshade Spectrophotometer*)	LED alone promoted long-term perceptible bleaching but was not compatible with that of high-concentrated HP. The bleaching outcomes of violet irradiation to 37% CP were maintained over time, with LED/CP demonstrating comparable results to HP even after 12 months.
Mondelli 2018 [67]	RCT	Assess the impact of a hybrid light (HL) source on patients undergoing various in-office bleaching procedures in terms of color change, stability, and tooth sensitivity.	20 patients were divided into the following (split-mouth): Group 1: 35% Lase Peroxide Sensy (LPS) + HL with HP 35% Group 2: 35% LPS with HP 35% Group 3: 25% LPS + HL with HP 25% Group 4: 35% HP (WHP): HP 35% For the groups activated with HL, the HP was applied on the enamel surface three consecutive times using a 3 × 2 min protocol. For the other groups, HP was applied 3 × 15 min, totaling 45 min.	Color at baseline, 24 h, one week, and one, 12, and 36 months after (*Easyshade Spectrophotometer*). Tooth sensitivity immediately following treatment, 24 h, and one week after (*Visual Analog Scale*).	Statistical analysis showed no significant differences in ΔE between in-office bleaching techniques with or without HL at the evaluated times. The groups without HL showed significant ΔE differences between 24 h and later follow-up times, along with increased tooth sensitivity in the initial periods. All techniques and bleaching agents were effective in maintaining color stability over the 36-month evaluation.

**Table 2:** Studies focused on at-home dental bleaching included in this review [41,68,69,70,71,72,73,74,75,76,77,78].

Authors	Type of Study	Problem	Intervention and Control	Measurements	Outcomes
Sutil 2022 [41]	RCT	Assess the effectiveness of bleaching, tooth sensitivity, and gum irritation in whitening patients using 10% compared to 37% carbamide peroxide.	80 patients were allocated to the following: Group 1: 37% CP Group 2: 10% CP In both groups, patients performed whitening for 3 weeks, 4 h/day for 10% group and 30 min/day for 37% group.	Color at baseline, weekly, and 30 days after treatment (*Vita Classical*, *Vita Bleached guide*, *and Easyshade Spectrophotometer*). Tooth sensitivity and gingival irritation daily for three weeks throughout the treatment (*Numeric Rating Scale and Visual Analog Scale*).	The 37% CP group showed quicker whitening results than the 10% CP group within 1–3 weeks, although both groups had similar bleaching outcomes one month after treatment. There were no significant differences in sensitivity or gingival irritation between the 10% and 37% CP groups.
Pavani 2023 [68]	RCT	Examine changes in color and sensitivity of the teeth using 10% carbamide peroxide.	66 patients were allocated to the following: Group 1: trays for 2 h daily Group 2: trays for 4 h daily Group 3: trays for 8 h daily	Color at baseline, one, two, and three weeks after the beginning of the bleaching treatment, as well as two weeks after the treatment (*Vita Bleached guide and Easyshade Spectrophotometer*) Tooth sensitivity at baseline, one, two, and three weeks after the beginning of the bleaching treatment, as well as two weeks after the treatment (*Visual Analog Scale*).	Group 3 showed significantly higher color change in the upper arch than Group 1 in subjective analysis from T1 to T4. However, no statistical differences were found between the groups in objective analysis.
Chemin 2021 [69]	RCT	Evaluate different protocols for at-home use of 10% hydrogen peroxide in whitening effectiveness and tooth sensitivity.	72 patients were allocated to the following: Group 1: HP 10% once daily for 15 min Group 2: HP 10% once daily for 30 min Bleaching was performed for 14 days in both groups.	Color before bleaching, during bleaching (1st and 2nd weeks), and 1 month after the bleaching (*Vita Classical*, *Vita Bleached guide*, *and Easyshade Spectrophotometer*) Tooth sensitivity (*Numeric Rating Scale and Visual Analog Scale*).	After 2 weeks of bleaching, a significant whitening effect was observed across all color measurements, with no difference between G1 and G2. Both the absolute risk and intensity of tooth sensitivity were similar.
Chemin 2018 [70]	RCT	Evaluate the risk for and intensity of tooth sensitivity and color change of at-home dental bleaching with 4% and 10% hydrogen peroxide.	78 patients were allocated to the following: Group 1: 4%HP twice daily for 30 min Group 2: 10%HP twice daily for 30 min Bleaching was performed for 14 days in both groups.	Color before bleaching, during bleaching (1st and 2nd weeks), and 1 month after the bleaching (*Vita Classical*, *Vita Bleached guide*, *and Easyshade Spectrophotometer*) Tooth sensitivity (*Numeric Rating Scale and Visual Analog Scale*).	The group using HP 10 had a higher absolute risk and intensity of tooth sensitivity than the HP 4 group. Both groups showed significant whitening after one month and no difference between them.
Botelho 2017 [71]	RCT	Investigate the effectiveness of two home bleaching modalities on whitening of tetracycline-stained teeth.	26 patients were allocated to the following: Group 1: tray containing CP 15% 2 h or overnight during the 3-month trial period Group 2: strip containing HP 6.0% twice daily for 30 min during the 3-month trial period	Color: Lightness (L*), redness (a*), and yellowness (b*) were measured with colorimeter at baseline, one, two, and three months.	During a three-month period, a 6.0% hydrogen peroxide strip achieved comparable results to the 15% carbamide peroxide tray system.
Aka 2017 [72]	RCT	Compare the bleaching efficacy of two different at-home bleaching systems on teeth of different shades and their color stability after a 6-month follow-up.	92 patients were allocated to the following: Group 1: negative control Group 2: tray containing CP 10% 8–10 h daily for 14 days Group 3: pre-loaded tray containing HP 6% 1 h daily for 14 days	Color at the baseline, 10 days and 14 days of bleaching, 2 weeks, and 6 months after bleaching (*Vita Classical*, *Vita Bleached guide*, *and Easyshade Spectrophotometer*).	Both bleaching agents resulted in a bleaching effect, but 10% CP/PF was more potent.
Darriba 2019 [73]	RCT	Determine whether prolonging the daytime at-home bleaching treatment by 1 week increases the bleaching effect without causing more side effects.	50 participants were randomly divided into two groups: Group 1: CP 10% once daily for 2 h/14-day treatment Group 2: CP 10% once daily for 2 h/21-day treatment	Color at baseline, at the end of treatment, and 1 and 6 months afterward (*Vita Classical*, *Vita Bleached guide*, *and Easyshade Spectrophotometer*). Tooth sensitivity and Gingival irritation daily self-reported.	A 3-week daytime application of at-home bleaching achieves greater whitening results than a 2-week application, both immediately after treatment and at 1- and 6-months post-treatment, though it may cause slightly more side effects. Extending the treatment duration from 2 to 3 weeks is recommended for better and more lasting results.
De Geus 2017 [74]	RCT	Evaluate the color longevity after 30 months of at-home bleaching with CP 10% in smokers and nonsmokers.	60 patients, 30 smokers (Group 1) and 30 nonsmokers (Group 2) were subjected to bleaching with 10% CP for three hours daily for three weeks	Color at baseline and at one month and 30 months after dental bleaching (*Vita Classical*, *Vita Bleached guide*, *and Easyshade Spectrophotometer*).	For both shade guides, only the assessment time factor was statistically significant. Both groups showed effective whitening at baseline, which remained stable at one month. However, after 30 months, color rebound was noted in both groups.
Llena 2020 [75]	Retrospective study	Assess the whitening efficacy of a 16% carbamide peroxide gel after 42 months of clinical follow-up.	95 patients CP 16% was applied for 90 min a day for 4 weeks using individualized trays.	Color at baseline, 1 week after the end of treatment, and every 6 months until completing 42 months of follow-up (*Easyshade Spectrophotometer*).	The home application of 16% CP gel for 90 min a day for 4 weeks using individualized trays resulted in whitening that remained stable over the 42 months of follow-up.
Mailart 2021 [76]	RCT	Compare the bleaching efficacy of HP 10% at-home systems using PT or CT (prefill or customize trays).	60 participants were allocated to the following: Group 1: HP 10% PT-30 min daily Group 2: HP 10% CT-30 min daily Group 3: CP 10% CT-2 h daily Group 4: CP 10% CT-8 h daily Bleaching was performed for 14 days in both groups.	Color (*Vita Classical*, *Vita Bleached guide*, *and Easyshade Spectrophotometer*). Tooth sensitivity at 2, 7, and 14 days after the beginning of treatment and 48 h after the end of treatments (*Visual Analog Scale*) and gingival condition at baseline, at the end of the treatment, and 48h after the end (*Löe index*).	After 1 year, color differences among the groups were similar, as was the sensitivity risk. Sensitivity intensity and gingival irritation were mild across all gels.
Martini 2021 [77]	RCT	Evaluate the color change stability and patient satisfaction after one year of at-home bleaching with 10% carbamide peroxide in trays with or without reservoirs.	46 patients were allocated to the following: Group 1: tray with reservoirs Group 2: tray without reservoirs CP 10% (3 h/daily; 21 days)	Color at baseline, one month, and one year after the completion of bleaching (*Vita Classical*, *Vita Bleached guide*, *and Easyshade Spectrophotometer*).	Bleaching tray design had no impact on bleaching stability with 10% CP, and patients were highly satisfied with results regardless of tray design. No significant color rebound was observed one year after bleaching with 10% CP.
Pinto 2017 [78]	RCT	Assess the colorimetric variation in the incisors and canines of adolescents aged 12 to 20 years undergoing at-home whitening, as well as examine tooth sensitivity during the procedures using a questionnaire.	30 adolescents were allocated to the following: Group 1: HP 6.0% (1 h a day for 7 days) Group 2: HP 7.5% (1 h a day for 7 days) Group 3: HP 10% (30 min twice a day for 7 days) Group 4: Control group—placebo (1 h a day for 7 days).	Color at baseline, 7, 30, 180, and 360 days after treatment (*Vita Bleached guide*).	Both tooth whitening gels and whitening strips produced similar results one month post-treatment. All products showed color stability after 12 months of follow-up.

**Table 3:** Studies focused on combined dental bleaching included in this review [79,80,81,82].

Authors	Type of Study	Problem	Intervention and Control	Measurements	Outcomes
Takamizawa 2023 [79]	RCT	Assess the whitening effectiveness, as well as the intensity and absolute risk of tooth sensitivity, during dual whitening when prefilled at-home whitening trays are used between in-office whitening sessions.	66 subjects were divided into the following: Group 1: At-home whitening was performed 10 times between the in-office whitening treatments (once a day for 90 min). Group 2: At-home whitening was performed 5 times between the in-office whitening treatments (once a day for 90 min). Group 3: Only in-office whitening was performed. An in-office whitening agent containing HP 35% (20 min/3 times at weekly intervals). A prefilled tray with a whitening agent containing HP 6% was used for at-home whitening.	Color before and after treatment six times (*Spectrophotometer*). Tooth sensitivity during and up to 24 h after whitening (*Visual Analog Scale*).	Dual whitening may lead to quicker and more intense whitening results compared to in-office whitening alone.
Zhong 2023 [80]	Clinical trial	Compare the clinical effectiveness of at-home, in-office, and combined bleaching treatments.	41 participants were divided into the following: Group 1: at-home bleaching using CP 10% for 14 days Group 2: two sessions of in-office bleaching using HP 40% with a one-week interval Group 3: one session of in-office bleaching followed by at-home bleaching for 7 days Group 4: at-home bleaching for 7 days followed by one session of in-office bleaching	Color at baseline, 8, 15, and 43 after the end of the bleaching treatment (*Vita Classical*, *Vita Bleached guide*, *and Easyshade Spectrophotometer*). Tooth sensitivity was recorded for 16 days (*Visual Analog Scale*).	All bleaching treatments showed significant color improvement, with similar color changes observed across different regimens at all evaluation time points. The order of treatments, whether in-office or at-home bleaching, did not influence the whitening effectiveness. However, in-office and combined bleaching regimens resulted in greater tooth sensitivity compared to at-home bleaching.
Pinto 2019 [81]	Clinical trial	Assess dental bleaching methods that use hydrogen peroxide, focusing on tooth sensitivity and gingival irritation.	75 patients were divided into the following: Group 1: at-home 10%HP for 15 days of continuous use (1 h per day) Group 2: in-office HP 40% in three sessions (40 min each session) Group 3: combined with one initial session with HP 40% and the rest with HP 10% for 15 days of continuous use.	Color at baseline, after 24 h, and 8 and 15 days from the end of treatment (*Vita Classical*, *Vita Bleached guide*, *and Easyshade Spectrophotometer*). Tooth sensitivity and gingival irritation at baseline, after 24 h, and 8 and 15 days from the end of treatment (*Visual Analog Scale*).	All the dental bleaching techniques proved equally effective in promoting tooth color change. The at-home and the combined techniques may cause greater dental sensitivity than the in-office technique and led to a higher prevalence of gingival irritation.
Rodrigues 2018 [82]	Clinical trial	Assess the impact of combining at-home and in-office bleaching treatments on tooth sensitivity and whitening effectiveness.	41 patients were divided into the following: Group 1: in-office bleaching with HP 38% (45 min) + second session Group 2: in-office bleaching with HP 38% + tray containing CP 10% (4 h daily) delivered during 7 consecutive days	Color at 7 days after each in-office session (for patients receiving in-office procedures only) or after the end of at-home bleaching (for the combined protocol) and 6 months after the last procedure for both bleaching protocols (*Vita Classical*, *Vita Bleached guide*, *and Easyshade Spectrophotometer*). Tooth sensitivity	After one in-office bleaching session, there was no difference in bleaching effectiveness and tooth sensitivity between performing a second in-office session and associating it with 1-week at-home bleaching.

**Table 4:** Studies focused on in-office versus at-home dental bleaching included in this review [80,81].

Authors	Type of Study	Problem	Intervention and Control	Measurements	Outcomes
Mounika 2018 [83]	Clinical trial	Compare the clinical performance, longevity, and associated tooth sensitivity of two vital bleaching methods (in-office and at-home bleaching) using a split-mouth design.	30 patients were divided into the following: Group 1: HP 35% 3 × 15 min with an interval of 7 days between each session Group 2: CP 16%-night guard bleaching 8 h daily for 3 weeks	Color at 1-, 2-, 3-, and 4-week intervals during bleaching and postoperatively at 3- and 6-month intervals (*Vita Classical*, *Vita Bleached guide*, *and Easyshade Spectrophotometer*). Tooth sensitivity (*Visual Analog Scale*).	At-home and in-office bleaching treatments are equally effective in achieving tooth whitening. However, color evaluation at 3 and 6 months revealed a greater color decline with the in-office bleaching procedure.
Moghadam 2013 [84]	RCT	Assess the color change, rebound effect, and sensitivity associated with at-home bleaching using 15% carbamide peroxide and power bleaching with 38% hydrogen peroxide.	20 patients were divided into the following: Group 1: at-home CP 15% 4 h/day, 2 weeks Group 2: in-office dental bleaching HP 38% for 33 × 15 min	Color before bleaching, immediately after bleaching, at 2 weeks, 1-, 3-, and 6-month intervals (*Vita Classical*, *Vita Bleached guide*, *and Easyshade Spectrophotometer*). Tooth sensitivity (*Visual Analog Scale*).	Both bleaching techniques produced similar tooth-whitening results and post-operative sensitivity. However, power bleaching resulted in faster color regression, even though the color in both groups returned to baseline levels after 6 months.

**Table 5:** Risk of bias of in-office dental bleaching studies included in this review.

Articles	Adequate Sequence Generated	Allocation Concealment	Blinding	Incomplete Outcome Data	Registration Outcome Data
Altınışık 2023 [56]	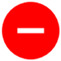	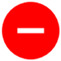	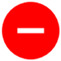	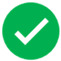	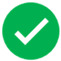
Brugnera 2020 [57]	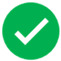	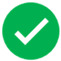	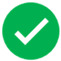	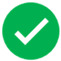	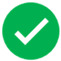
Martins 2018 [58]	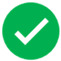	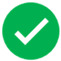	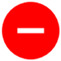	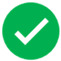	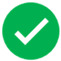
Bersezio 2019 [59]	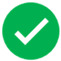	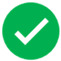	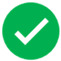	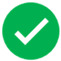	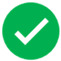
Favoreto 2024 [60]	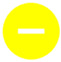	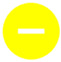	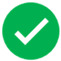	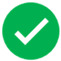	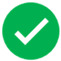
Mayer-Santos 2022 [61]	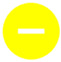	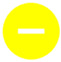	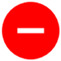	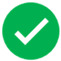	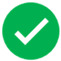
Tsujimoto 2021 [62]	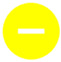	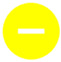	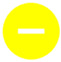	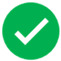	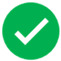
Santos 2021 [63]	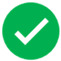	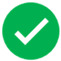	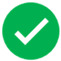	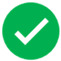	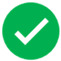
Sobral 2021 [64]	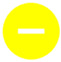	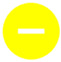	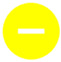	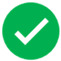	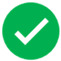
Estay 2020 [65]	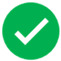	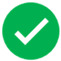	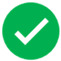	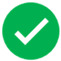	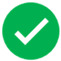
Kury 2022 [66]	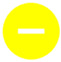	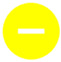	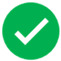	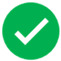	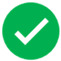
Mondelli 2018 [67]	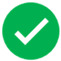	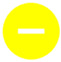	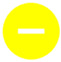	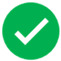	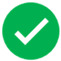

**Table 6:** Risk of bias of at-home dental bleaching studies included in this review.

Articles	Adequate Sequence Generated	Allocation Concealment	Blinding	Incomplete Outcome Data	Registration Outcome Data
Sutil et al., 2022 [41]	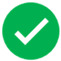	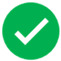	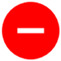	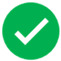	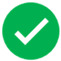
Pavani et al., 2023 [68]	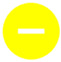	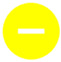	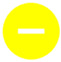	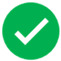	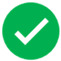
Chemin et al., 2021 [69]	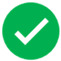	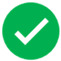	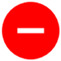	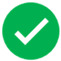	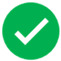
Chemin et al., 2018 [70]	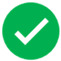	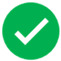	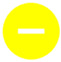	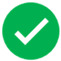	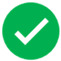
Botelho et al., 2017 [71]	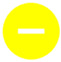	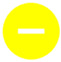	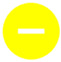	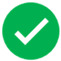	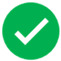
Aka et al., 2017 [72]	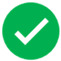	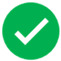	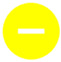	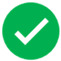	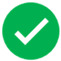
Darriba et al., 2019 [73]	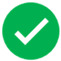	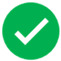	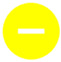	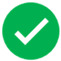	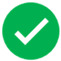
De Geus et al., 2017 [74]	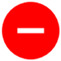	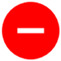	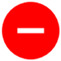	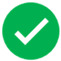	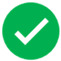
Llena et al., 2020 [75]	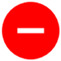	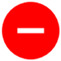	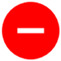	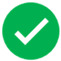	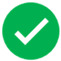
Mailart et al., 2021 [76]	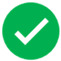	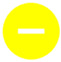	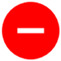	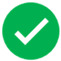	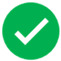
Martini et al., 2021 [77]	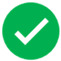	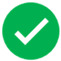	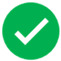	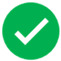	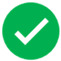
Pinto et al., 2017 [78]	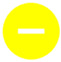	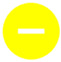	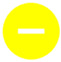	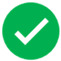	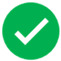

**Table 7:** Risk of bias of combined dental bleaching studies included in this review.

**Articles**	**Adequate Sequence Generated**	**Allocation Concealment**	**Blinding**	**Incomplete Outcome Data**	**Registration Outcome Data**
Takamizawa et al., 2023 [79]	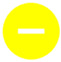	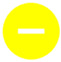	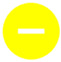	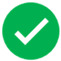	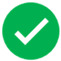
Zhong et al., 2023 [80]	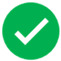	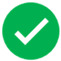	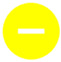	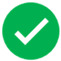	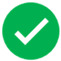
Pinto et al., 2019 [81]	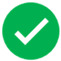	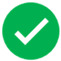	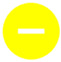	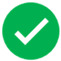	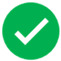
Rodrigues et al., 2018 [82]	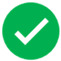	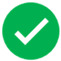	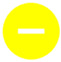	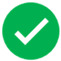	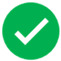

**Table 8:** Risk of bias of in-office versus at-home dental bleaching studies included in this review.

Articles	Adequate Sequence Generated	Allocation Concealment	Blinding	Incomplete Outcome Data	Registration Outcome Data
Mounika et al., 2018 [83]	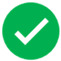	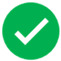	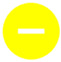	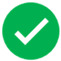	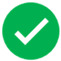
Moghadam et al., 2013 [84]	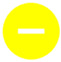	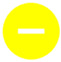	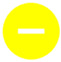	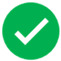	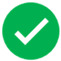

## Data Availability

The data presented in this study are available upon request from the corresponding author.
